# 3-[4-(Dimethyl­amino)benzyl­ideneamino]benzonitrile

**DOI:** 10.1107/S1600536809011568

**Published:** 2009-04-02

**Authors:** Hai-Jun Xu, Qin-Ying Tan, Li-Jing Cui, Kun Qian

**Affiliations:** a Ordered Matter Science Research Center, College of Chemistry and Chemical Engineering, Southeast University, Nanjing 210096, People’s Republic of China; b JiangXi University of Traditional Medicine, Nanchang 330047, People’s Republic of China

## Abstract

The mol­ecule of the title Schiff base, C_16_H_15_N_3_, is non-planar and displays a *trans* configuration with respect to the C=N double bond. The two benzene rings make a dihedral angle of 49.24 (3)°.

## Related literature

For general background on Schiff base coordination complexes, see: Garnovskii *et al.* (1993 ▶). For a related structure, see: Gong & Xu (2008 ▶)
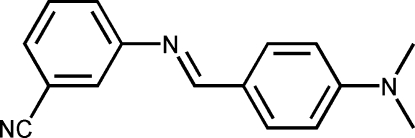


## Experimental

### 

#### Crystal data

C_16_H_15_N_3_
*M* *_r_* = 249.31
Monoclinic, 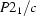

*a* = 6.0924 (6) Å
*b* = 29.127 (3) Å
*c* = 7.3768 (7) Å
β = 92.924 (1)°
*V* = 1307.3 (2) Å^3^
*Z* = 4
Mo *K*α radiation
μ = 0.08 mm^−1^
*T* = 293 K
0.30 × 0.20 × 0.20 mm

#### Data collection

Bruker SMART CCD area-detector diffractometer
Absorption correction: multi-scan (*CrystalClear*; Rigaku, 2005 ▶) *T*_min_ = 0.961, *T*_max_ = 1.000 (expected range = 0.947–0.985)
7018 measured reflections
2559 independent reflections
2241 reflections with *I* > 2σ(*I*)
*R*_int_ = 0.064

#### Refinement

*R*[*F* ^2^ > 2σ(*F* ^2^)] = 0.039
*wR*(*F* ^2^) = 0.110
*S* = 1.05
2559 reflections
174 parameters
H-atom parameters constrained
Δρ_max_ = 0.19 e Å^−3^
Δρ_min_ = −0.25 e Å^−3^

### 

Data collection: *SMART* (Bruker, 2000 ▶); cell refinement: *SAINT* (Bruker, 2000 ▶); data reduction: *SAINT*; program(s) used to solve structure: *SHELXS97* (Sheldrick, 2008 ▶); program(s) used to refine structure: *SHELXL97* (Sheldrick, 2008 ▶); molecular graphics: *SHELXTL* (Sheldrick, 2008 ▶); software used to prepare material for publication: *SHELXL97*.

## Supplementary Material

Crystal structure: contains datablocks I, global. DOI: 10.1107/S1600536809011568/dn2436sup1.cif

Structure factors: contains datablocks I. DOI: 10.1107/S1600536809011568/dn2436Isup2.hkl

Additional supplementary materials:  crystallographic information; 3D view; checkCIF report

## Figures and Tables

**Fig. 1.**
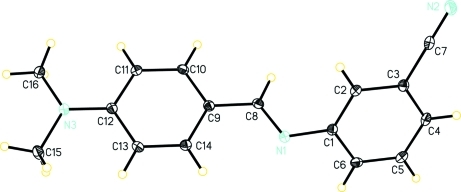
A view of the title compound with the atomic numbering scheme. Displacement ellipsoids are drawn at the 30% probability level.

## supplementary crystallographic information

### Comment

Schiff base compounds have attracted great attention and been extensively investigated in terms of their crystallography and coordination chemistry (Garnovskii *et al.*, 1993). As a continuation of our studies on Schiff-base compounds, we here report the synthesis and crystal structure of the title compound (I).

The molecule displays a *trans* configuration with respect to the C=N double bond (Fig. 1). The values of the C—C, C=C, C—N and C=N bond distances in (I) are similar to the corresponding bond lengths in 4-(2-Hydroxybenzylideneamino)benzonitrile(Gong & Xu, 2008). The molecule is nonplanar and the dihedral angle between the planes of the two benzene rings is 49.24 (0.03) °.

### Experimental

All chemicals were obtained from commercial sources and directly used without further purification. 3-aminobenzonitrile (1.18 g, 10 mmol) and 4-(dimethylamino)benzaldehyde (1.49 g, 10 mmol) were dissolved in ethanol (20 ml). The mixture was heated to reflux for 6 h, then cooled to room temperature overnight and large amounts of a yellow precipitate were formed. Yellow crystals were obtained by recrystallization from ethanol(yield:2.04,82%). ^1^H-NMR(CDCl_3_, 300 MHz): δ3.10 (s, 6 H), 6.73–6.76(d, 2 H), 7.45–7.50 (m, 4 H), 7.84–7.87 (d, 2 H), 8.27 (s, 1 H). Esi-MS: calcd for C_16_H_15_N_3_*m*/*z *249.31, found 250.18[*M*+1]. For the X-ray diffraction analysis, suitable single crystals of compound (I) were obtained after two weeks by slow evaporation from an ethanol solution.

### Refinement

All H atoms attached to C were positioned geometrically and treated as riding, with C—H = 0.93 (aromatic), 0.93 (methine) or 0.96 Å (methyl) with *U*_iso_(H) = 1.2Ueq(C).

### Crystal data

**Table:**

C_16_H_15_N_3_	*F*(000) = 528
*M**_r_* = 249.31	*D*_x_ = 1.267 Mg m^−^^3^
Monoclinic, *P*2_1_/*c*	Mo *K*α radiation, λ = 0.71073 Å
Hall symbol: -P 2ybc	Cell parameters from 2559 reflections
*a* = 6.0924 (6) Å	θ = 2.8–26.0°
*b* = 29.127 (3) Å	µ = 0.08 mm^−^^1^
*c* = 7.3768 (7) Å	*T* = 293 K
β = 92.924 (1)°	Block, yellow
*V* = 1307.3 (2) Å^3^	0.30 × 0.20 × 0.20 mm
*Z* = 4	

### Data collection

**Table:**

Bruker SMART CCD area-detector diffractometer	2559 independent reflections
Radiation source: fine-focus sealed tube	2241 reflections with *I* > 2σ(*I*)
graphite	*R*_int_ = 0.064
φ and ω scans	θ_max_ = 26.0°, θ_min_ = 2.8°
Absorption correction: multi-scan (*CrystalClear*; Rigaku, 2005)	*h* = −7→7
*T*_min_ = 0.961, *T*_max_ = 1.000	*k* = −35→25
7018 measured reflections	*l* = −9→8

### Refinement

**Table:**

Refinement on *F*^2^	Primary atom site location: structure-invariant direct methods
Least-squares matrix: full	Secondary atom site location: difference Fourier map
*R*[*F*^2^ > 2σ(*F*^2^)] = 0.039	Hydrogen site location: inferred from neighbouring sites
*wR*(*F*^2^) = 0.110	H-atom parameters constrained
*S* = 1.05	*w* = 1/[σ^2^(*F*_o_^2^) + (0.0596*P*)^2^ + 0.1755*P*] where *P* = (*F*_o_^2^ + 2*F*_c_^2^)/3
2559 reflections	(Δ/σ)_max_ < 0.001
174 parameters	Δρ_max_ = 0.19 e Å^−^^3^
0 restraints	Δρ_min_ = −0.25 e Å^−^^3^

### Special details

**Table:**

Geometry. All e.s.d.'s (except the e.s.d. in the dihedral angle between two l.s. planes) are estimated using the full covariance matrix. The cell e.s.d.'s are taken into account individually in the estimation of e.s.d.'s in distances, angles and torsion angles; correlations between e.s.d.'s in cell parameters are only used when they are defined by crystal symmetry. An approximate (isotropic) treatment of cell e.s.d.'s is used for estimating e.s.d.'s involving l.s. planes.
Refinement. Refinement of *F*^2^ against ALL reflections. The weighted *R*-factor *wR* and goodness of fit *S* are based on *F*^2^, conventional *R*-factors *R* are based on *F*, with *F* set to zero for negative *F*^2^. The threshold expression of *F*^2^ > σ(*F*^2^) is used only for calculating *R*-factors(gt) *etc*. and is not relevant to the choice of reflections for refinement. *R*-factors based on *F*^2^ are statistically about twice as large as those based on *F*, and *R*- factors based on ALL data will be even larger.

### Fractional atomic coordinates and isotropic or equivalent isotropic displacement parameters (Å^2^)

**Table:**

	*x*	*y*	*z*	*U*_iso_*/*U*_eq_	
N1	0.61108 (15)	0.27979 (3)	0.59956 (13)	0.0190 (2)	
N2	0.28428 (16)	0.47022 (3)	0.70360 (14)	0.0263 (3)	
N3	0.14557 (15)	0.08098 (3)	0.61116 (13)	0.0189 (2)	
C1	0.65373 (17)	0.32723 (4)	0.58571 (14)	0.0171 (2)	
C2	0.50894 (17)	0.36071 (4)	0.64200 (14)	0.0169 (2)	
H2A	0.3790	0.3523	0.6941	0.020*	
C3	0.56051 (17)	0.40708 (4)	0.61955 (14)	0.0175 (3)	
C4	0.75672 (18)	0.42049 (4)	0.54437 (14)	0.0190 (3)	
H4A	0.7889	0.4514	0.5285	0.023*	
C5	0.90207 (18)	0.38678 (4)	0.49403 (15)	0.0207 (3)	
H5A	1.0341	0.3951	0.4452	0.025*	
C6	0.85265 (17)	0.34081 (4)	0.51572 (15)	0.0197 (3)	
H6A	0.9532	0.3186	0.4833	0.024*	
C7	0.40682 (18)	0.44200 (4)	0.66975 (15)	0.0198 (3)	
C8	0.42252 (17)	0.26531 (4)	0.53867 (14)	0.0174 (3)	
H8A	0.3245	0.2863	0.4847	0.021*	
C9	0.35526 (17)	0.21769 (4)	0.55017 (14)	0.0171 (3)	
C10	0.14627 (17)	0.20363 (4)	0.48467 (15)	0.0188 (3)	
H10A	0.0515	0.2251	0.4298	0.023*	
C11	0.07713 (17)	0.15861 (4)	0.49941 (15)	0.0187 (3)	
H11A	−0.0617	0.1502	0.4526	0.022*	
C12	0.21502 (17)	0.12527 (4)	0.58471 (14)	0.0162 (2)	
C13	0.42785 (17)	0.13953 (4)	0.64815 (14)	0.0176 (3)	
H13A	0.5238	0.1182	0.7028	0.021*	
C14	0.49460 (17)	0.18430 (4)	0.63022 (14)	0.0175 (2)	
H14A	0.6355	0.1926	0.6722	0.021*	
C15	0.30017 (19)	0.04552 (4)	0.67163 (16)	0.0229 (3)	
H15A	0.3783	0.0553	0.7812	0.034*	
H15B	0.4027	0.0401	0.5793	0.034*	
H15C	0.2218	0.0177	0.6943	0.034*	
C16	−0.06638 (18)	0.06583 (4)	0.53393 (16)	0.0222 (3)	
H16A	−0.1766	0.0881	0.5599	0.033*	
H16B	−0.1035	0.0368	0.5860	0.033*	
H16C	−0.0590	0.0626	0.4049	0.033*	

### Atomic displacement parameters (Å^2^)

**Table:**

	*U*^11^	*U*^22^	*U*^33^	*U*^12^	*U*^13^	*U*^23^
N1	0.0189 (5)	0.0166 (5)	0.0216 (5)	0.0000 (4)	0.0009 (4)	0.0003 (4)
N2	0.0295 (5)	0.0194 (5)	0.0304 (6)	−0.0017 (4)	0.0058 (4)	−0.0035 (4)
N3	0.0188 (5)	0.0154 (5)	0.0224 (5)	−0.0007 (4)	0.0011 (4)	0.0008 (4)
C1	0.0179 (5)	0.0166 (5)	0.0162 (5)	−0.0016 (4)	−0.0034 (4)	0.0017 (4)
C2	0.0150 (5)	0.0187 (6)	0.0170 (5)	−0.0024 (4)	0.0005 (4)	0.0002 (4)
C3	0.0190 (5)	0.0182 (6)	0.0151 (5)	−0.0014 (4)	−0.0010 (4)	−0.0016 (4)
C4	0.0211 (5)	0.0184 (6)	0.0172 (5)	−0.0060 (4)	−0.0022 (4)	0.0005 (4)
C5	0.0161 (5)	0.0262 (6)	0.0196 (6)	−0.0049 (5)	0.0002 (4)	0.0005 (5)
C6	0.0149 (5)	0.0231 (6)	0.0209 (5)	0.0023 (4)	−0.0010 (4)	−0.0014 (4)
C7	0.0225 (6)	0.0170 (6)	0.0199 (5)	−0.0056 (5)	0.0009 (4)	−0.0009 (4)
C8	0.0165 (5)	0.0171 (6)	0.0187 (5)	0.0033 (4)	0.0027 (4)	0.0015 (4)
C9	0.0171 (5)	0.0170 (5)	0.0174 (6)	0.0012 (4)	0.0039 (4)	0.0001 (4)
C10	0.0164 (5)	0.0180 (6)	0.0221 (6)	0.0042 (4)	0.0013 (4)	0.0018 (4)
C11	0.0147 (5)	0.0196 (6)	0.0219 (6)	−0.0004 (4)	0.0014 (4)	−0.0001 (4)
C12	0.0181 (5)	0.0162 (5)	0.0148 (5)	0.0015 (4)	0.0046 (4)	−0.0003 (4)
C13	0.0186 (5)	0.0176 (5)	0.0167 (5)	0.0041 (4)	0.0013 (4)	0.0013 (4)
C14	0.0162 (5)	0.0189 (5)	0.0175 (5)	0.0008 (4)	0.0012 (4)	−0.0007 (4)
C15	0.0292 (6)	0.0158 (6)	0.0233 (6)	0.0000 (5)	−0.0039 (5)	0.0009 (4)
C16	0.0198 (6)	0.0184 (6)	0.0285 (6)	−0.0020 (4)	0.0035 (5)	−0.0007 (5)

### Geometric parameters (Å, °)

**Table:**

N1—C8	1.2833 (14)	C8—H8A	0.9300
N1—C1	1.4105 (14)	C9—C10	1.3998 (15)
N2—C7	1.1466 (15)	C9—C14	1.4013 (15)
N3—C12	1.3746 (14)	C10—C11	1.3834 (16)
N3—C15	1.4526 (14)	C10—H10A	0.9300
N3—C16	1.4531 (14)	C11—C12	1.4109 (15)
C1—C2	1.3924 (15)	C11—H11A	0.9300
C1—C6	1.3984 (15)	C12—C13	1.4181 (15)
C2—C3	1.3986 (15)	C13—C14	1.3743 (15)
C2—H2A	0.9300	C13—H13A	0.9300
C3—C4	1.3988 (15)	C14—H14A	0.9300
C3—C7	1.4434 (16)	C15—H15A	0.9600
C4—C5	1.3854 (16)	C15—H15B	0.9600
C4—H4A	0.9300	C15—H15C	0.9600
C5—C6	1.3836 (16)	C16—H16A	0.9600
C5—H5A	0.9300	C16—H16B	0.9600
C6—H6A	0.9300	C16—H16C	0.9600
C8—C9	1.4501 (15)		
			
C8—N1—C1	117.49 (9)	C14—C9—C8	121.43 (10)
C12—N3—C15	120.76 (9)	C11—C10—C9	121.69 (10)
C12—N3—C16	120.20 (9)	C11—C10—H10A	119.2
C15—N3—C16	116.99 (9)	C9—C10—H10A	119.2
C2—C1—C6	119.09 (10)	C10—C11—C12	120.71 (10)
C2—C1—N1	122.86 (10)	C10—C11—H11A	119.6
C6—C1—N1	118.04 (10)	C12—C11—H11A	119.6
C1—C2—C3	119.44 (10)	N3—C12—C11	121.91 (9)
C1—C2—H2A	120.3	N3—C12—C13	120.73 (10)
C3—C2—H2A	120.3	C11—C12—C13	117.33 (10)
C2—C3—C4	121.21 (10)	C14—C13—C12	121.08 (10)
C2—C3—C7	119.83 (10)	C14—C13—H13A	119.5
C4—C3—C7	118.94 (10)	C12—C13—H13A	119.5
C5—C4—C3	118.64 (10)	C13—C14—C9	121.54 (10)
C5—C4—H4A	120.7	C13—C14—H14A	119.2
C3—C4—H4A	120.7	C9—C14—H14A	119.2
C6—C5—C4	120.59 (10)	N3—C15—H15A	109.5
C6—C5—H5A	119.7	N3—C15—H15B	109.5
C4—C5—H5A	119.7	H15A—C15—H15B	109.5
C5—C6—C1	120.95 (10)	N3—C15—H15C	109.5
C5—C6—H6A	119.5	H15A—C15—H15C	109.5
C1—C6—H6A	119.5	H15B—C15—H15C	109.5
N2—C7—C3	177.67 (13)	N3—C16—H16A	109.5
N1—C8—C9	123.00 (10)	N3—C16—H16B	109.5
N1—C8—H8A	118.5	H16A—C16—H16B	109.5
C9—C8—H8A	118.5	N3—C16—H16C	109.5
C10—C9—C14	117.61 (10)	H16A—C16—H16C	109.5
C10—C9—C8	120.95 (10)	H16B—C16—H16C	109.5
